# Melatonin Is Involved in Regulation of Bermudagrass Growth and Development and Response to Low K^+^ Stress

**DOI:** 10.3389/fpls.2017.02038

**Published:** 2017-11-28

**Authors:** Liang Chen, Jibiao Fan, Zhengrong Hu, Xuebing Huang, Erick Amombo, Ao Liu, Aoyue Bi, Ke Chen, Yan Xie, Jinmin Fu

**Affiliations:** ^1^Key Laboratory of Plant Germplasm Enhancement and Specialty Agriculture, Wuhan Botanical Garden, Chinese Academy of Sciences, Wuhan, China; ^2^College of Animal Science and Technology, Yangzhou University, Yangzhou, China; ^3^University of Chinese Academy of Sciences, Beijing, China; ^4^College of Resources and Environmental Science, South-Central University for Nationalities, Wuhan, China

**Keywords:** bermudagrass, melatonin, LK stress, K^+^ content, photosystem II (PSII) activity

## Abstract

Melatonin (*N*-acetyl-5-methoxytryptamine) plays critical roles in plant growth and development and during the response to multiple abiotic stresses. However, the roles of melatonin in plant response to K^+^ deficiency remain largely unknown. In the present study, we observed that the endogenous melatonin contents in bermudagrass were remarkably increased by low K^+^ (LK) treatment, suggesting that melatonin was involved in bermudagrass response to LK stress. Further phenotype analysis revealed that exogenous melatonin application conferred Bermudagrass enhanced tolerance to LK stress. Interestingly, exogenous melatonin application also promoted bermudagrass growth and development at normal condition. Furthermore, the K^+^ contents measurement revealed that melatonin-treated plants accumulated more K^+^ in both shoot (under both control and LK condition) and root tissues (under LK condition) compared with those of melatonin non-treated plants. Expression analysis indicated that the transcripts of K^+^ transport genes were significantly induced by exogenous melatonin treatment in bermudagrass under both control and LK stress conditions, especially under a combined treatment of LK stress and melatonin, which may increase accumulation of K^+^ content profoundly under LK stress and thereby contributed to the LK-tolerant phenotype. In addition, we investigated the role of melatonin in the regulation of photosystem II (PSII) activities under LK stress. The chlorophyll fluorescence transient (OJIP) curves were obviously higher in plants grown in LK with melatonin (LK+Mel) than those of plants grown in LK medium without melatonin application for 1 or 2 weeks, suggesting that melatonin plays important roles in PSII against LK stress. After a combined treatment of LK stress and melatonin, the values for performance indexes (PI_ABS_, PI_Total_, and PI_CS_)_,_ flux ratios (φP_0_, ΨE_0_, and φE_0_) and specific energy fluxes (ET_O_/RC) were significantly improved compared with those of LK stress alone, suggesting that melatonin plays positive roles in protecting PSII activity under LK stress. Collectively, this study reveals an important role of melatonin in regulating bermudagrass response to LK stress.

## Introduction

Potassium is an essential element for plant growth and development, and a vital determinant of crop yield and quality. Potassium (K^+^) also plays crucial roles in many fundamental processes in living plant cells, such as osmoregulation, electrical neutralization, regulation of membrane potential, cotransport of sugars, and so on (Clarkson and Hanson, 1980; Xu et al., 2006; Wang and Wu, 2013). The concentration of K^+^ in the cytoplasm is approximately 100 mM, while it varies from 10 to 200 mM in the vacuoles (Walker et al., 1996). Although the K^+^ concentration is high in plant cells, it is limited in soils, typically within the micromolar range (0.1–1 mM) (Schroeder et al., 1994; Maathuis, 2009). Therefore, plants evolved a series of mechanisms, including physiological, biochemical, and morphological alterations, to respond to K^+^-deficiency stress (Schachtman and Shin, 2007; Wang and Wu, 2013; Wang et al., 2016).

Under low-K^+^ (LK) conditions, plants absorb K^+^ through the high-affinity K^+^ transporters and some K^+^ channels (Maathuis and Sanders, 1997; Véry et al., 2014). In Arabidopsis, the AtHAK5 transporter (Rubio et al., 2000; Nieves-Cordones et al., 2010) and AKT1 channel (Lagarde et al., 1996; Hirsch et al., 1998; Spalding et al., 1999; Ivashikina et al., 2001) expressed primarily in the roots, were identified as the two major K^+^ uptake components, which mediate K^+^ absorption in roots under LK conditions (Gierth et al., 2005; Pyo et al., 2010; Nieves-Cordones et al., 2014). *AtHAK5* gene transcription was remarkably induced by K^+^ deficiency, while AKT1 was regulated mainly at the posttranslational level (Gierth et al., 2005; Xu et al., 2006).

Melatonin (*N*-acetyl-5-methoxytryptamine) was initially identified as an important animal hormone, which regulates sleep, mood, sexual behavior, circadian rhythms, and anti-oxidative activities (Tan et al., 2003; Reiter et al., 2009; Galano et al., 2011; Escames et al., 2012; Venegas et al., 2012; Calvo et al., 2013). Later studies revealed that melatonin is also ubiquitously presented in plants and unicellular organisms (Dubbels et al., 1995; Van Tassel et al., 1995; Hardeland and Poeggeler, 2003). Melatonin is widely involved in various aspects of plant growth and development, including flowering, circadian rhythms, leaf senescence, root system architecture (Arnao and Hernández-Ruiz, 2009; Byeon et al., 2012; Pelagio-Flores et al., 2012; Tan et al., 2012; Wang et al., 2013; Zhang et al., 2014). Tan et al. (2012) reported that the root system and production were greater in the melatonin-treated corn plants than those of the non-melatonin-treated plants (Tan et al., 2012). Likewise, melatonin promoted soybean growth and increased seed production (Wei et al., 2015). In addition, another important role of melatonin is related to abiotic and biotic stresses response, including salt, drought, extreme temperature, and pathogen infection (Zhang et al., 2013; Bajwa et al., 2014; Lee et al., 2014; Shi and Chan, 2014; Shi et al., 2015a,b,c). The role of melatonin involvement in LK stress response, however, remains largely unknown.

Potassium is a primary, essential nutrient for turfgrass production. A deficiency in K confers bermudagrass reduced winter hardiness, decreased disease resistance and diminished rhizome and stolon production (Miller, 1999). The present study revealed that the endogenous melatonin contents in bermudagrass were remarkably increased under LK condition. Further phenotype analysis revealed that exogenous melatonin application conferred Bermudagrass not only enhanced tolerance to LK stress, but also promoted bermudagrass growth and development under normal condition. Furthermore, melatonin-treated plants accumulated more K^+^ in both shoot (under both control and LK condition) and root tissues (under LK condition) compared with those of melatonin non-treated plants. Expression analysis indicated that the transcripts of K^+^ transport genes were significantly induced by exogenous melatonin treatment in bermudagrass under both control and LK stress conditions, especially under a combined treatment of LK stress and melatonin. In addition, the role of melatonin in the regulation of photosystem II (PSII) activities under LK stress was further investigated. Collectively, this study reveals an important role of melatonin in regulating bermudagrass response to LK stress.

## Materials and Methods

### Plant Materials and Growth Conditions

Bermudagrass ‘WBD128’ collected from Xiaojiang City, Zhejiang Province, China, was used in this study. Bermudagrass seeds were surface-sterilized with 75% ethanol for 1 min followed by 10% NaClO for 3 min then washed with sterile water for four times. The sterilized bermudagrass seeds were sown on MS medium containing 1.5% sucrose, pH 5.8. After stratification at 4°C for 3 days in darkness, the MS plates were transferred to the growth chamber (16 h light/8 h dark, 26°C) for seed germination.

Uniform stolons of the genotype ‘WBD128’ were planted in plastic pots, which were kept in the greenhouse for 1 month to establish under growth conditions of 12 h light/12 h dark, 28°C.

### Treatment

For LK stress treatment, 6-day-old bermudagrass plants on MS plate were transferred to LK or MS medium with different concentrations of melatonin and treated for the indicated times described in the figure legends section, and then the fresh weight and primary root length were recorded. The LK medium was modified from MS medium according to the method described previously (Xu et al., 2006). Briefly, 2.99 mM CaCl_2_ and 1.25 mM KH_2_PO_4_ were replaced by 2.99 mM Ca(NO_3_)_2_ and 1.25 mM NH_4_H_2_PO_4_, 18.79 mM KNO_3_ and 20.6 mM NH_4_NO_3_ were removed, and 1.5 mM MgSO_4_ was unchanged. The final K^+^ concentration in LK agar medium was adjusted by adding KCl. In the present study, the K^+^ concentration was 100 μM in the LK medium.

For analysis of low K^+^ stress on the PSII activities of bermudagrass, the established ‘WBD128’ plants were transferred to LK or MS liquid medium with or without melatonin for the indicated times described in figure legends.

### Chlorophyll (Chl) a Fluorescence Transient

The pulse-amplitude modulation (PAM) fluorometer (PAM 2500, Heinz Walz GmbH) was employed to measure chlorophyll (Chl) a fluorescence transient. The measurement was performed as described previously (Chen et al., 2014; Hu et al., 2016). After the collected bermudagrass leaves were pre-adapted in the dark for 30 min, the OJIP transients were triggered by red light (3,000 μmol photons m^-2^ s^-1^) to guarantee closure of all reaction centers of PS II and to evaluate a real fluorescence intensity. The Chl a fluorescence emission induced by the strong light pulses was subsequently tested and digitized between 10 μs and 320 ms. The OJIP transient data was processed using the JIP-test as reported by Chen et al. (2014).

### Quantification of Melatonin

The measurement of endogenous melatonin contents was performed by enzyme-linked immunosorbent assay (ELISA) as previously described with some modifications (Fan et al., 2015; Hu et al., 2016). Briefly, a 0.3 g of leaf tissues was ground into fine powder in liquid nitrogen, transferred into tubes containing 5 ml of extraction solution (Acetone:Methanol:Water = 89:10:1), and then homogenized on ice for 1 h. After centrifugation at 4500 × *g* for 5 min at 4°C, the supernatant was transferred to a new tube containing 0.5 ml of 1% trichloric acid. The mixture was centrifuged at 4500 × *g* for 10 min at 4°C, and the extract was used for quantification of endogenous melatonin using the Melatonin ELISA Kit (EK-DSM; Buhlmann Laboratories AG, Schonenbuch, Switzerland) according to the manufacturer’s instruction.

### Measurement of K^+^ Content

After growth on MS or LK medium for 2 weeks, shoots and roots of the bermudagrass seedlings were harvested for K^+^ content measurements. The plant samples were washed thoroughly with distilled water, placed in oven at 105°C for 30 min to deactivate the enzymes, and then dried at 80°C for 48 h. After weighing, the samples were ground into a fine power, and then digested in a solution (HClO_4_:HNO_3_ = 1:4, v/v). Subsequently, the K^+^ content assays were performed using ICP-MS (Thermo Fisher Scientific, Waltham, MA, United States) as previously described (Xie et al., 2017).

### RNA Isolation and Quantitative Real-Time PCR

The total RNA was isolated from the bermudagrass roots using TRIzol reagent (Invitrogen, United States) according to the manufacturer’s instruction. DNaseI was used to remove any contaminated genomic DNA, and then a 2 μg of total RNA was reversely transcribed into cDNAs using M-MLV reverse transcriptase (Promega, United States). Real-time quantitative RT-PCR was performed by the method described previously by Chen et al. (2012), and the *CdACT2* gene was used as a quantitative control. The specific primer sequences were listed in **Table 1**.

**Table 1:** Primers used in this study.

Primer name	Primer sequence (5′ to 3′)
CdACT2 F	5′- TCTGAAGGGTAAGTAGAGTAG -3′
CdACT2 R	5′- ACTCAGCACATTCCAGCAGAT -3′
CdHAK9 F	5′- TTCGTGAGGCTGGACGCGTCG -3′
CdHAK9 R	5′- CTGCCGTTCGTGGCGCGCACC -3′
Potassium transporter 1 (CdKT1) F	5′- TACGAGTCGTCGGTGGACGGG -3′
Potassium transporter 1 (CdKT1) R	5′- ATCTGGAACCGGACCCTGCGC -3′
Potassium transporter 23 (CdKT23) F	5′- GACGACTCGGCCGCGGGCGCG -3′
Potassium transporter 23 (CdKT23) R	5′- GCCGTAGACAACACCGAGGGT -3′

### Statistical Analysis

All experiments were performed with at least three repetitions. The significance of differences was determined by ANOVA or Student’s *t*-test (*P* < 0.05), as indicated in the figure legends.

## Results

### The Endogenous Melatonin Contents under LK Stress

**FIGURE 1:**
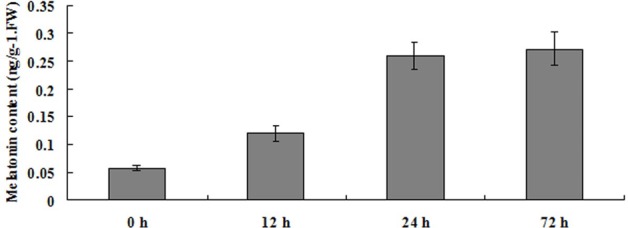
Changes of endogenous melatonin concentration in response to LK stress in Bermudagrass. Six-day-old bermudagrass plants on MS plate were transferred to LK medium for 0, 12, 24, and 72 h. The experiments were repeated three times. Data shown are the mean ± SD.

To investigate the role of melatonin in plants response to LK stress, the endogenous melatonin production in bermudagrass leaves was measured after being treated with LK stress for 12, 24, and 72 h. The results revealed that the endogenous melatonin contents were remarkably increased for 2- to 4.7-fold during the 12–72 h of LK treatment (**Figure 1**). The significant increase of endogenous melatonin content by LK stress suggests that melatonin may be involved in plant response to LK stress in bermudagrass.

### Melatonin Involvement in Plant Growth and Development and LK Stress Response

**FIGURE 2:**
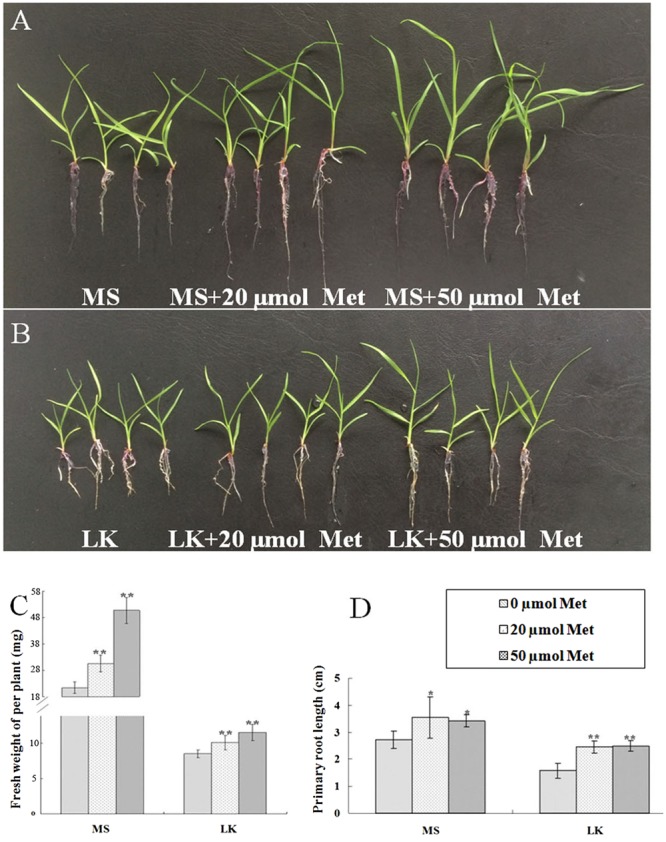
Exogenous melatonin application promoted growth and development of bermudagrass and conferred bermudagrass improved tolerance to LK stress. Six-day-old bermudagrass plants were transferred to MS **(A)** or LK agar plates **(B)** with different concentrations of melatonin for another 2 weeks. **(C)** Quantification of the fresh weight and **(D)** primary root length as shown in **(A)** and **(B)**. Data shown are the mean ± SD (*n* = 3). ^∗∗^*P* < 0.01, ^∗^*P* < 0.05 by Student’s *t-*test.

**FIGURE 3:**
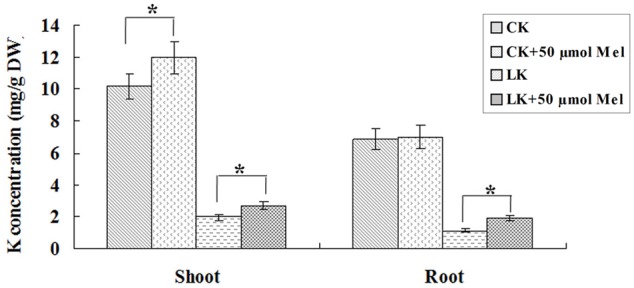
Effects of melatonin treatment on K^+^ contents in roots and shoots under MS or LK stress. The K^+^ content was measured after the plants were transferred to MS or LK medium with or without 50 μm melatonin. Data shown are the mean ± SD (*n* = 3). ^∗^*P* < 0.05 by Student’s *t-*test.

To further investigate the possible role of melatonin involvement in plant growth and development and LK stress response, we examined the phenotypic changes of bermudagrass under normal (MS) and LK stress conditions with or without melatonin treatment. The 6-day-old seedlings were transferred and vertically cultured on MS or LK medium containing 20 or 50 μm melatonin for 1 week. It was found that under both MS and LK conditions, melatonin-treated plants grew better as characterized by greater fresh weight and higher root length compared with those of non-melatonin-treated plants (**Figures 2A–D**). The primary root length of 20 or 50 μm melatonin-treated plants (MS+20 or 50 μm melatonin) was 130 and 125% of those of non-treated plants, while under LK condition, the root length of melatonin-treated plants were increased to 156 and 158% of those of non-treated plants (**Figures 2A–D**), suggesting that melatonin was involved in regulating both plant growth and development and LK response. In addition, the K^+^ contents of bermudagrass plants were measured after they were grown on MS or LK medium with or without 50 μm melatonin application. Under LK condition, melatonin-treated plants accumulated more K^+^ in both shoot and root tissues compared with those of melatonin non-treated plants. By contrast, when plants were grown on MS medium, the higher K^+^ accumulation in the shoots of melatonin-treated plants were observed compared with those of melatonin non-treated plants, whereas there was no significant difference in K^+^ content in roots between melatonin and non-melatonin treated plants (**Figure 3**). The results suggest that the greater K^+^ content accumulation in melatonin-treated plants may contribute to the LK-tolerant phenotype and improved growth and development of bermudagrass.

**FIGURE 4:**
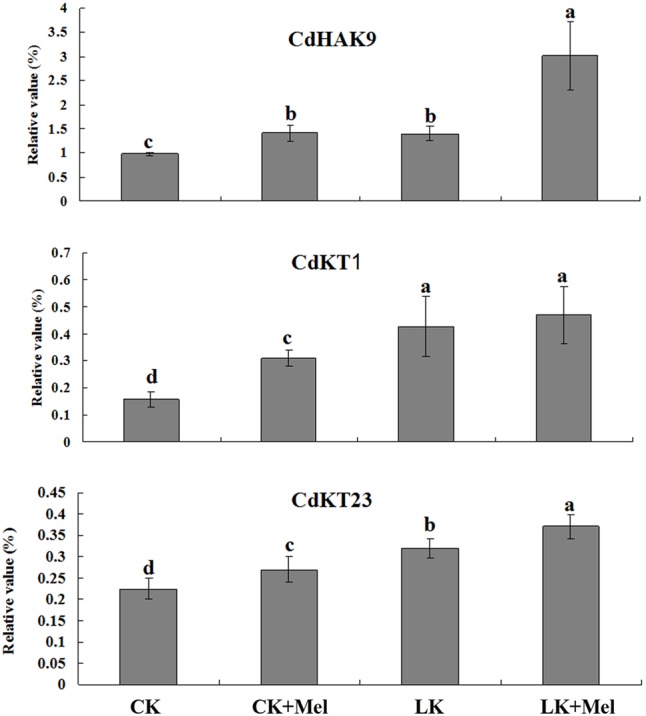
Effects of melatonin on the expression of genes involved in K^+^ transport. Six-day-old bermudagrass plants were transferred to MS or LK medium with or without 50 μm melatonin for 48 h, and then total RNAs isolated from roots and shoots were used for quantitative RT-PCR analysis. Data shown are the mean ± SD (*n* = 3). Different letters indicate significantly different values (*P* < 0.05 by Tukey’s test).

Next, the expression of genes involved in potassium transport was investigated by RT-PCR. The results revealed that expression levels of the three tested gene were significantly induced by LK stress or melatonin treatment, and further increased to higher levels after LK stress and melatonin combined treatment (**Figure 4**). These results suggested that the higher K^+^ content accumulation in melatonin-treated plants may result from the increased expression of K^+^ transport genes.

### Effects of Low K^+^ Stress on the Photosystem II (PSII) Activities of Bermudagrass

**FIGURE 5:**
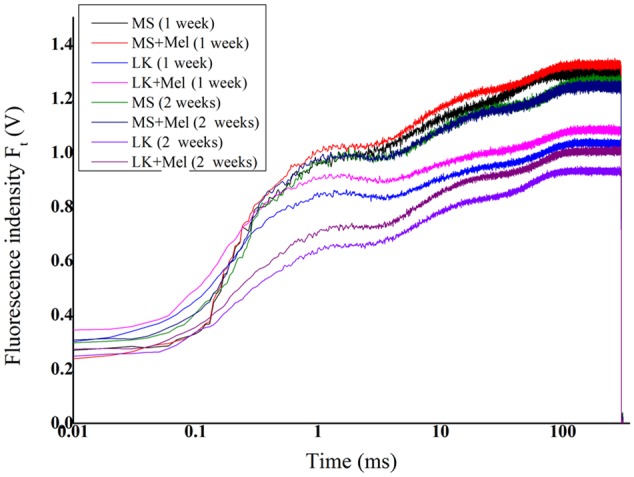
Changes of chlorophyll fluorescence transients (OJIP curve) under different treatments.

To investigate whether melatonin is involved in regulation of PSII activities under LK stress, OJIP fluorescence transient curves were tested. As shown in **Figure 5**, the OJIP fluorescence transients were significantly affected by LK stress. The OJIP curves dropped remarkably after LK treatment compared with those of plants that were grown in control condition (MS liquid medium). There were no significant differences in the OJIP transient curves between plants grown in MS medium with or without melatonin application for 1 week or 2 weeks. However, the OJIP curves were obviously higher in plants grown in LK with melatonin (LK+Mel) than those of plants grown in LK medium without melatonin application for 1 or 2 weeks (**Figure 5**), suggesting that melatonin played important roles in PSII against LK stress. Besides, the OJIP curves of plants grown in LK medium for 2 weeks were significantly lower than those of plants grown in LK medium for 1 week, revealing that PSII activity was further decreased during plants exposure to LK stress for prolonged days.

**FIGURE 6:**
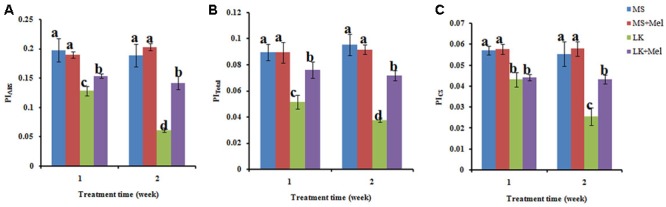
Changes of performance index (PI) as deduced by JIP-test analysis of fluorescence transients. **(A)** PI_ABS_, PI for energy conservation from exciton to the reduction of the intersystem electron; **(B)** PI_Total_, PI for energy conservation from exciton to the reduction of PSI end acceptors; **(C)** PI_CS_, PI on a cross section basis. Data shown are the mean ± SD (*n* = 3). Different letters indicate significantly different values (*P* < 0.05 by Tukey’s test).

**FIGURE 7:**
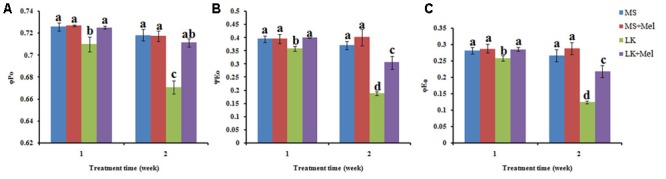
Changes of flux ratios as deduced by JIP-test analysis of fluorescence transients. **(A)** φP_0_, the maximum quantum yield of PSII photochemistry; **(B)** ΨE_0_, efficiency/probability with which a PSII trapped electron is transferred from Q_A_ to Q_B_; **(C)** φE_0_, the quantum yield of the electron transport flux from Q_A_ to Q_B_. Data shown are the mean ± SD (*n* = 3). Different letters indicate significantly different values (*P* < 0.05 by Tukey’s test).

**FIGURE 8:**
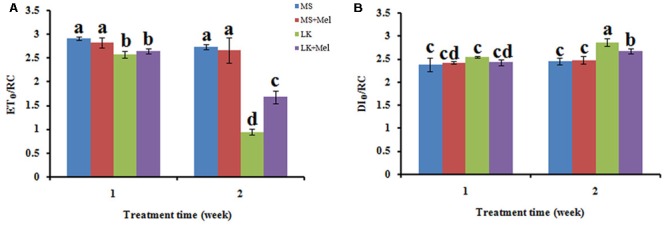
Changes of specific energy fluxes per active PSII reaction center (RC) as deduced by JIP-test analysis of fluorescence transients. **(A)** ET_O_/RC, electron transport flux (further than QA^-^) per RC; **(B)** DI_O_/RC, energy fluxes for dissipation.

The JIP-test was further applied to study the photosynthetic behavior of PSII. The results showed that the photosynthetic behavior of PSII was significantly different between plants treated with or without melatonin under low K^+^ stress, but not under control condition (MS) with or without exogenous melatonin (**Figures 6–8**). In brief, the structural and functional parameters of photosynthetic behavior including performance indexes, flux ratios and specific energy fluxes were improved in melatonin-treated plants under LK condition.

The performance indexes (PI) such as PI_ABS_, PI_Total_, and PI_CS_ are widely used to describe the overall activity of PSII. As shown in **Figure 6**, the values of PI_ABS_, PI_Total_, and PI_CS_ were all significantly lower in plants treated by LK stress than those of plants grown under MS condition. For instance, the value of PI_ABS_ reduced remarkably after LK treatment for 1 week, and it was further decreased after LK for 2 weeks. However, it was reversed by melatonin application during LK treatment (**Figure 6**). Similar results were also observed in the parameters, such as PI_Total_ and PI_CS_ (**Figure 6**). These results suggested that exogenous melatonin played a protective role in PSII activity under LK stress.

For the flux ratios, φP_0_ (maximum quantum yield of primary photochemistry) decreased significantly under LK stress, and it further declined as the LK treatment time prolonged. After melatonin application during LK stress, the parameter of φP_0_ was higher than that of LK treatment (**Figure 7**). Similarly, the parameters of other flux ratios such as ΨE_0_ (Efficiency/probability with which a PSII trapped electron is transferred from Q_A_ to Q_B_) and φE_0_ (quantum yield of the electron transport flux from Q_A_ to Q_B_) displayed the alteration pattern similar to φP_0_ (**Figure 7**). These results imply that melatonin plays positive roles in improving the behavior in both electron donor side and acceptor side of PSII when plants were treated by LK stress. The parameters of specific energy fluxes, including ET_O_/RC [Electron transport flux (further than QA^-^) per RC] and DI_O_/RC (Energy fluxes for dissipation) were further calculated. The ET_O_/RC value was lower in the plants of LK treatment than that of MS treatment, and a slight but not significant increase was observed in the plants treated with melatonin when the plants were treated for 1 week (**Figure 8**). After the plants were treated for 2 weeks, a remarkable decrease of ET_O_/RC was observed in the plants by LK treatment, and melatonin mitigated the damage effectively (**Figure 8**). On the contrary, DI_O_/RC increased slightly in the plants after they were treated by LK stress for 1 week. When the plants were treated for 2 weeks, DI_O_/RC showed the highest value under LK condition among four different treatments, while DI_O_/RC decreased in LK+Mel regime (**Figure 8**).

## Discussion

Recently, several studies have revealed that the functions of melatonin are related to many aspects of plant growth and development. The most frequently mentioned functions of melatonin are its roles in abiotic and biotic stresses such as drought (Shi et al., 2015a; Wei et al., 2015), salt (Wei et al., 2015), extreme temperature (Bajwa et al., 2014; Shi and Chan, 2014; Shi et al., 2015b,c), and bacterial infection (Lee et al., 2014; Shi et al., 2015b). However, so far little is known about the roles of melatonin involved in LK stress response. Only one recent study reported that melatonin was involved in the absorption of K^+^ elements by plants under stress conditions (Li et al., 2016).

In this study, we revealed at least three important issues. Firstly, exogenous melatonin application promoted bermudagrass growth and development. Wei et al. (2015) demonstrated that melatonin has significant potential for enhancement of soybean growth and seed production. Similarly, the root system was larger in melatonin-treated corn plants than that of non-melatonin-treated corn plants, and thus the estimated production of the melatonin-treated corn was 20% greater than that from the non-melatonin-treated plants (Tan et al., 2012). Consistent with previous investigations, we found that primary root length and fresh weight were larger in melatonin-treated bermudagrass plants than those of non-melatonin-treated plants.

Secondly, the endogenous melatonin contents were remarkably increased after LK treatment, and exogenous melatonin application conferred bermudagrass improved LK stress tolerance. Previous studies have reported that the endogenous melatonin contents were increased when plants were exposed to abiotic stress, such as cold and heat (Shi and Chan, 2014; Shi et al., 2015c). Therefore, we investigated whether endogenous melatonin concentration was enhanced in LK-treated plants. Meaningfully, our results revealed that the endogenous melatonin contents in bermudagrass were remarkably increased after LK treatment, suggesting the possible involvement of melatonin in plant response to LK stress in bermudagrass. Furthermore, exogenous melatonin application conferred bermudagrass improved LK stress tolerance. The measurement of K^+^ contents and expression analysis of K^+^ transport genes revealed that under LK stress, the melatonin-treated plants increased expression of K^+^ transport genes, resulted in the greater K^+^ content accumulation, and thereby contributed to the LK-tolerant phenotype. Next step, it is meaningful to investigate how the melatonin regulates the expression of K^+^ transport genes.

Thirdly, melatonin is involved in regulation of PSII activities under LK stress. Chlorophyll *a* fluorescence has been widely employed to investigate plant photosystem activity especially under abiotic stress conditions, and it can be as an indicator to evaluate the ability of plant tolerance to abiotic stress (Chen et al., 2014; Fan et al., 2015; Hu et al., 2016). As an essential nutrient element, K^+^ significantly affects many fundamental processes, including photosynthesis and assimilation product transport or translocation, and thereby determines plant growth and development (Wang et al., 2013). However, little is known in detail of how LK stress regulates bermudagrass PSII activity, and whether melatonin is involved in regulation of PSII activities under LK stress. In the present study, our results revealed that the OJIP curves dropped remarkably after LK treatment compared with those of plants grown under control condition, revealing that the LK stress resulted in the damage to bermudagrass PSII. By contrast, the OJIP curves were obviously increased in plants grown in LK medium with melatonin application (LK+Mel), suggesting that melatonin plays important roles in PSII against LK stress. Our previous studies also revealed that melatonin plays essential roles in PSII against cold stress (Fan et al., 2015; Hu et al., 2016). These results consistently implied the crucial roles of melatonin involvement in PSII regulation under abiotic stress.

The JIP-test was further employed to quantify the photosynthetic behavior of plants based on structural and functional parameters. The performance index PI _total_, which represented energy conservation from exciton to the reduction of PSI end acceptors, incorporated several parameters from OJIP, and thus is a sensitive parameter of the JIP-test for assessing plant photochemical activities under abiotic stress condition (Clark et al., 2000; Yusuf et al., 2010; Chen et al., 2013; Liu et al., 2016). Here it was found that melatonin greatly improved the PI_Total_ value under LK stress, suggesting that melatonin plays positive roles in maintaining the photochemical activities under LK stress. The flux ratios, such as φP_0_, φE_0_, and ΨE_0_ could evaluate the degree of injury of PSII components. In the present study, LK stress significantly decreased the behavior of φP_0_, φE_0_, and ΨE_0_, revealing the damage to PSII caused by LK stress. After melatonin application during LK stress, these parameters were significantly improved compared with those of LK stress, implying that melatonin plays positive roles in improving the behavior in both electron donor side and acceptor side of PSII under LK stress.

## Conclusion

Our study showed that LK stress can result in the enhancement of endogenous melatonin content in bermudagrass. Furthermore, exogenous melatonin application not only promoted bermudagrass growth and development under normal condition but also conferred bermudagrass enhanced tolerance to LK stress. The enhanced LK stress tolerance by melatonin can be attributed to longer primary root, greater K^+^ content accumulation due to improved expression of K^+^ transport genes and increased PSII activities.

## Author Contributions

LC designed the research. LC, JFa, ZH, XH, AL, and AB performed experiments and data analysis. LC wrote the manuscript in close collaboration with all authors. EA, YX, JFu, and KC revised the manuscript. All authors contributed to numerous discussions and revised the manuscript.

## Conflict of Interest Statement

The authors declare that the research was conducted in the absence of any commercial or financial relationships that could be construed as a potential conflict of interest.
